# Endophytic *Streptomyces hygroscopicus* OsiSh-2-Mediated Balancing between Growth and Disease Resistance in Host Rice

**DOI:** 10.1128/mBio.01566-21

**Published:** 2021-08-10

**Authors:** Yan Gao, Qing Ning, Yuanzhu Yang, Ying Liu, Shuqi Niu, Xiaochun Hu, Huairong Pan, Zhigang Bu, Ning Chen, Jinyou Guo, Jinlan Yu, Lidan Cao, Peng Qin, Junjie Xing, Bin Liu, Xuanming Liu, Yonghua Zhu

**Affiliations:** a State Key Laboratory of Chemo/Biosensing and Chemometrics, Hunan Province Key Laboratory of Plant Functional Genomics and Developmental Regulation, College of Biology, Hunan University, Changsha, Hunan, China; b State Key Laboratory of Hybrid Rice, Yahua Seeds Science Academy of Hunan, Changsha, Hunan, China; c State Key Laboratory of Hybrid Rice, Hunan Hybrid Rice Research Center, Changsha, Hunan, China; Georgia Institute of Technology School of Biological Sciences

**Keywords:** endophytic actinobacteria, disease resistance, defense priming, fitness, proteomic analysis, photosynthetic efficiency

## Abstract

Plants fine-tune the growth-defense trade-off to survive when facing pathogens. Meanwhile, plant-associated microbes, such as the endophytes inside plant tissues, can benefit plant growth and stress resilience. However, the mechanisms for the beneficial microbes to increase stress resistance with little yield penalty in host plants remain poorly understood. In the present study, we report that endophytic Streptomyces hygroscopicus OsiSh-2 can form a sophisticated interaction with host rice, maintaining cellular homeostasis under pathogen-infection stress, and optimize plant growth and disease resistance in rice. Four-year field trials consistently showed that OsiSh-2 could boost host resistance to rice blast pathogen Magnaporthe oryzae while still maintaining a high yield. The integration of the proteomic, physiological, and transcriptional profiling analysis revealed that OsiSh-2 induced rice defense priming and controlled the expression of energy-consuming defense-related proteins, thus increasing the defense capability with the minimized costs of plant immunity. Meanwhile, OsiSh-2 improved the chloroplast development and optimally maintained the expression of proteins related to plant growth under pathogen stress, thus promoting the crop yield. Our results provided a representative example of an endophyte-mediated modulation of disease resistance and fitness in the host plant. The multilayer effects of OsiSh-2 implicate a promising future of using endophytic actinobacteria for disease control and crop yield promotion.

## INTRODUCTION

Global rice yield is under constant threat from various microbial pathogens (1). Breeding cultivars with resistance or application of resistance genes in rice is an efficient strategy to control diseases (2, 3). However, defense activity often comes at a yield penalty, a phenomenon known as the growth-defense trade-off (4, 5). For instance, rice lesion mimic mutants, including *spl24* (6) and *sles* (7), showed improved resistance to pathogen infection, while their agronomic traits were significantly weakened. Overexpression of the disease resistance genes, e.g., *PigmR* in rice, resulted in high and durable blast resistance, but a reduced yield was also observed (3). This growth and defense conflict was attributed to the reallocation of resources (8) or incompatibility of the regulating pathways between these two processes (9). Thus, integrative regulation of plant growth and disease resistance has become a major aim in crop breeding and currently received great attention (10). The roles of some host factors in balancing this trade-off have been recently reported, such as three growth-related transcription factors HBI1 in *Arabidopsis* (9); IPA1 and OsALDH2B1 in rice (11, 12); a DELLA-EDS1-mediated feedback regulatory loop in *Arabidopsis* (13), *Brassica* miR1885 (14), and potato miR160 (15); and plant hormones such as cytokinins, auxins, and abscisic acid (16, 17).

Plants can recruit beneficial microbes to improve plant growth, fitness, and stress resilience (18). Thus, the microorganisms which can mitigate the growth-defense trade-off are of great agricultural value. However, reports on these microbes are scarce. Until now, a well-studied example is *Trichoderma* (19), which showed biocontrol and plant growth-promoting capacities on plants, including melon (20), bean (21), and tomato (22). However, their regulation mechanisms on plant growth defense are so far not well established. Other potential candidates are endophytes within the plants. The specific habitat niche of endophytes allows them to better interact with plant physiological activities compared to the microorganisms derived from soil (23). It was reported that entophytic bacteria Pseudomonas sp. could promote shoots growth and resistance toward the soft rot disease of potato (24). The endophytic fungus Trichoderma asperellum could suppress vascular streak dieback incidence and promote side graft growth of Theobroma cacao (25).

One of the hallmark traits of microbial treatments is low cost. For instance, beneficial microbes could induce priming, a faster and stronger activation of cellular defense to pathogen challenge, in plants, resulting in enhanced stress resistance (26). Compared to sustaining a full-scale defense response, priming is an important strategy to allow the host to defend against pathogen attack in a timely and cost-efficient manner and thus can mitigate the dilemma posed by the defense-growth trade-off (27). The priming-inducing agents are increasingly considered for application in disease management. However, compared to the large body of research on rhizobacteria (28, 29), knowledge of endophytes as priming elicitors to stimulate defense response is still in its infancy. For example, the rice blast fungus Magnaporthe oryzae caused the most devastating rice disease. Only a few studies, e.g., an analysis of endophytic Pseudomonas putida BP25, have reported significant inhibition of *M. oryzae* by priming (30).

Our previous studies showed a rice endophyte Streptomyces hygroscopicus OsiSh-2 (here referred to as OsiSh-2) with excellent biocontrol activity against rice blast (31–33). In those studies, a series of experiments were done under greenhouse and field conditions. We disclosed that the symbiosis relationship of OsiSh-2 with rice might contribute to a simultaneous increase in rice blast resistance and yielding. Our results indicated that the colonization of OsiSh-2 in rice induced a priming response. Meanwhile, OsiSh-2 inoculation could improve the photosynthetic efficiency of the host, thus promoting rice growth. The comparative quantitative proteomic analysis of rice samples revealed the dynamic regulation of proteins involved in the growth and defense response processes in OsiSh-2-treated rice, thus optimizing the allocation of resources between growth and defense. These results confirmed the sophisticated OsiSh-2-mediated management, which led to a win-win situation between the growth and defense of rice.

## RESULTS AND DISCUSSION

### Endophytic OsiSh-2 enhances both disease resistance and yield of rice during *M. oryzae* infection.

**FIG 1:**
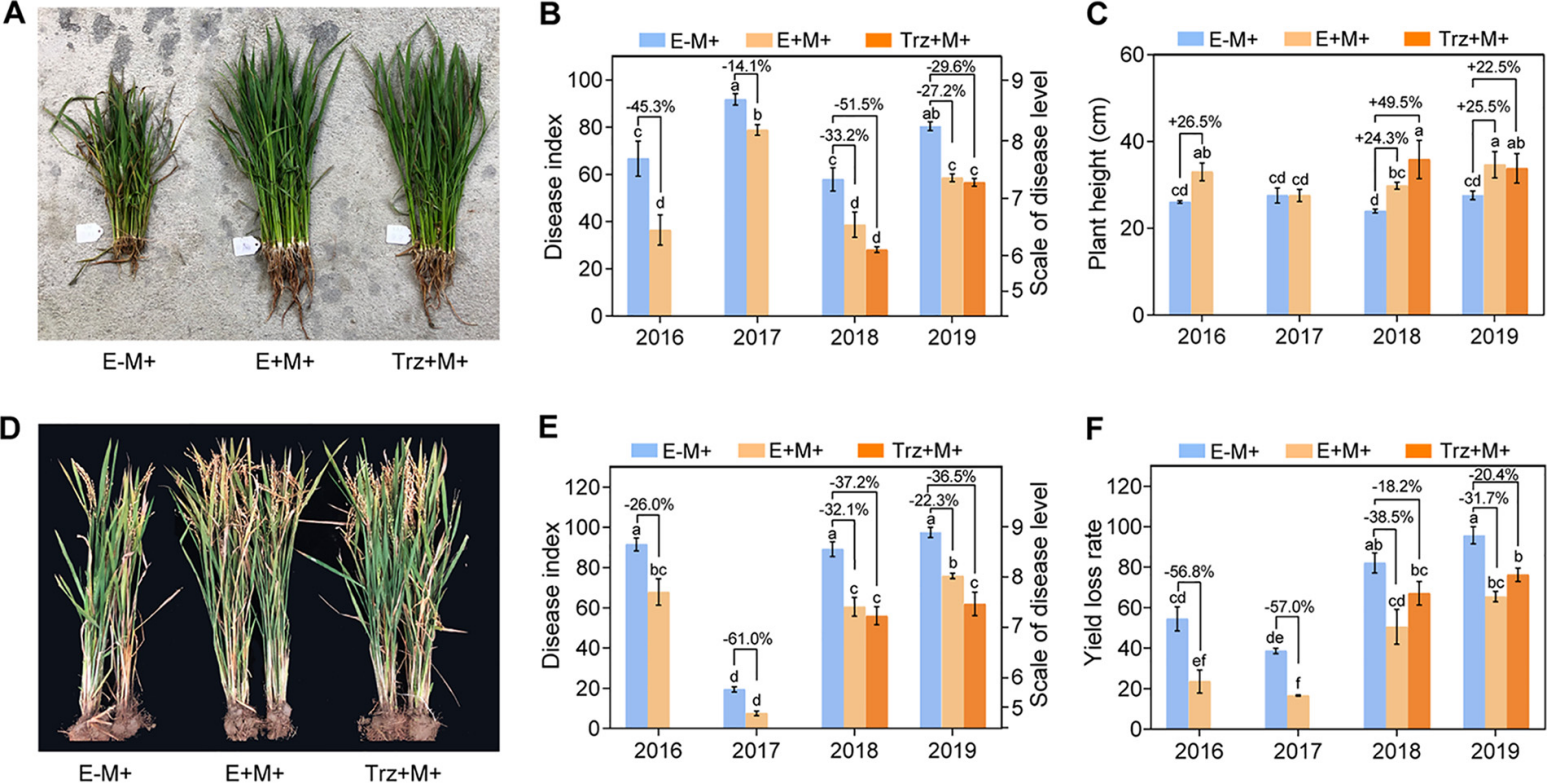
Streptomyces hygroscopicus OsiSh-2 enhances rice blast resistance and promoted rice growth in the field conditions. (A) Representative photograph of 1-month-old rice after the seedling blast in the year 2019 (15 plants per treatment). (B and C) Disease index (%) and plant height (cm) of 1-month-old rice after the seedling blast in the years 2016 to 2019. (D) Representative photograph of 4-month-old rice after the panicle blast in the year 2019 (two plants per treatment). (E and F) Disease index (%) and yield loss rate (%) of 4-month-old rice after the panicle blast in the years 2016 to 2019. E–M+, rice foliar presprayed with water; E+M+, rice foliar presprayed with OsiSh-2 spore suspension (10^7^ spores ml^−1^ in 2016 and 2017 and 10^8^ spores ml^−1^ in 2018 and 2019); Trz+M+, rice foliar presprayed with pesticide tricyclazole (1.5 μg/ml) after M. oryzae infection. Values are the means of two independent experiments performed each year, with three replicates per experiment, and 100 plants/replicate for the seedling blast and 20 plants/replicate for the panicle blast. Values are means ± standard deviation (SD). Bars with different letters are significantly different (ANOVA, *P* < 0.05) according to Duncan’s multiple-range test. Percentage changes followed by “+” or “–” above the bars were calculated by using the following formula: percent change = [(value of treated rice) – (value of untreated rice)]/(value of untreated rice) × 100%.

Our previous field experimental studies showed that OsiSh-2 could reduce the disease index in the seedling blast (33). To confirm that OsiSh-2 can steadily improve the disease control efficiency of host rice, we carried out another 4-year field trial in 2016 to 2019. To mimic the natural condition, the rice seedlings were surrounded by infected seedlings to allow the airborne spores to serve as inoculum. As shown in Fig. 1, OsiSh-2 foliar spraying treatment with *M. oryzae* infection (E+M+) significantly reduced the disease indexes of the seedling blast (Fig. 1A and B) and panicle blast (Fig. 1D and E) for up to 45.3 and 61.0%, respectively, compared to the OsiSh-2 untreated (E–M+) rice. Correspondingly, the seedling height increased up to 26.5% after the seedling blast (Fig. 1C), and the yield loss rate reduced up to 57.0% after the panicle blast (Fig. 1F).

In the year 2018 and 2019, we further complemented a positive-control treatment, i.e., pesticide tricyclazole (Trz+M+), one of the commonly used fungicides for rice blast disease. Interestingly, although Trz+M+ treatment showed a better disease control efficiency than E+M+ treatment with an average 9.7% lower disease index between 2018 and 2019 (Fig. 1E), the yield loss rate of the OsiSh-2 treatment was much lower (average 15.8%) than that of the tricyclazole (Fig. 1F). These results suggest a direct stimulation of rice growth by OsiSh-2, not just an indirect effect of the disease resistance, thereby alleviating the reduction of growth.

**FIG 2:**
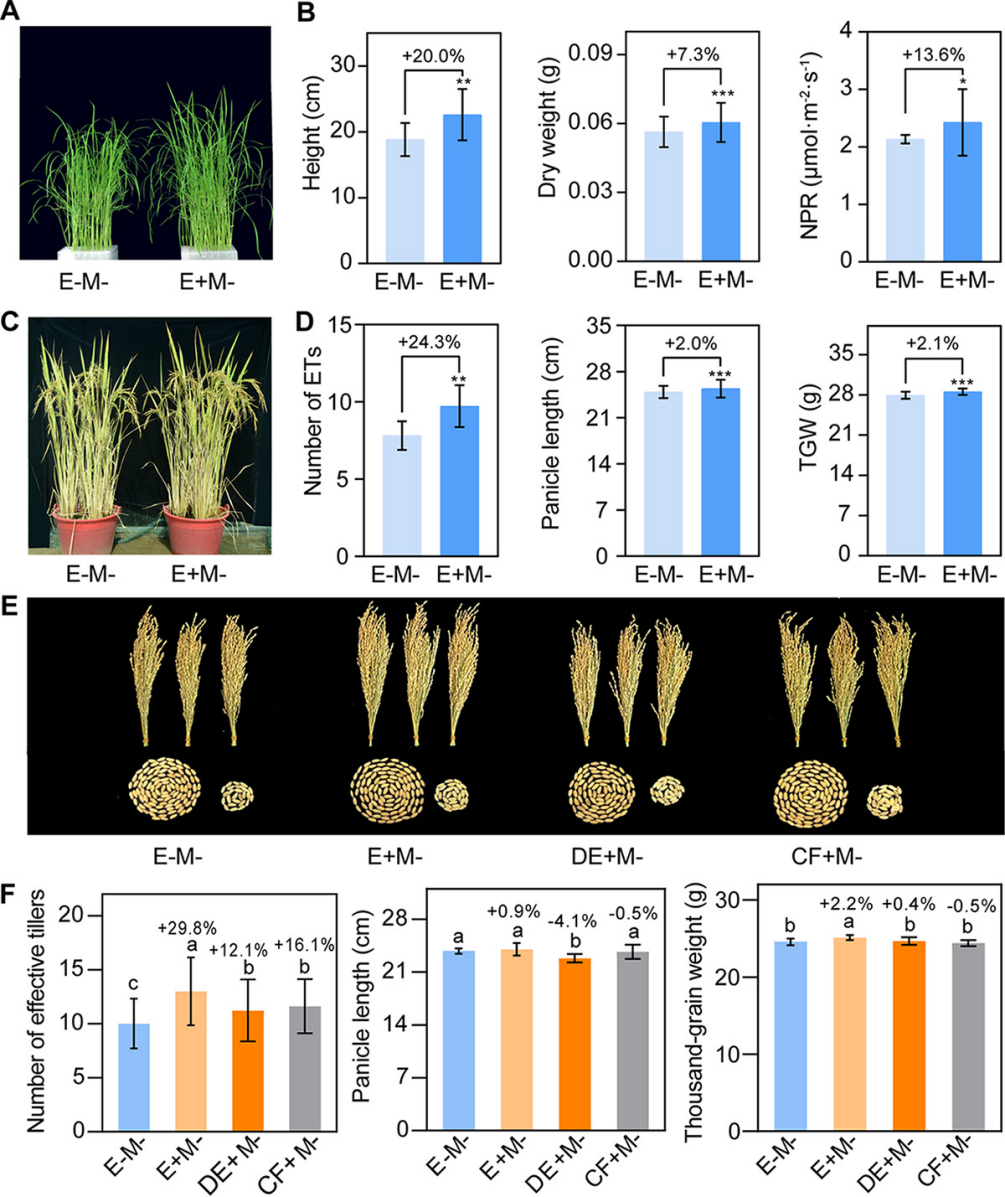
*S. hygroscopicus* OsiSh-2 promotes rice growth in greenhouse and field conditions. (A) Representative photograph of 28-day-old E–M– and E+M– rice without *M. oryzae* infection. (B) Plant height (cm) and net photosynthetic rate (NPR; μmol m^−2^ S^−1^) are used to represent the seedling growth traits. Values are means ± the SD (*n* = 50). (C) Representative photograph of 4-month-old E–M– and E+M– rice. (D) The numbers of effective tillers (ET) and the TGW (g) were determined to represent agronomic traits. (E) Representative photograph of all panicles in one plant (three plants per treatment) and all grains in a panicle (left, filled grains; right, flat grains) for E–M–, E+M–, and dead (DE+M–) OsiSh-2 and cell-free culture filtrate (CF+M–) rice. (F) The grain yield of rice in different treatments is presented as the number of effective tillers, the panicle length (cm), and the thousand-grain weight (g). All of the rice panicles were sampled. Values are means ± the SD (from 20 independent rice plants). Bars in panels B and C with asterisks are significantly different, as determined by the Tukey-Kramer test (*, *P* < 0.05; **, *P* < 0.01; ***, *P* < 0.001). Bars in panel F with different letters are significantly different (ANOVA, *P* < 0.05) according to Duncan’s multiple-range test. The percentage changes followed by “+” or “–” above the bars were calculated by using the following formula: percent change = [(value of treated rice) – (value of untreated rice)]/(value of untreated rice) × 100%.

To test whether OsiSh-2 conferred a desirable growth-promoting and yield-increases for its host rice, the growth parameters of E+M– and E–M– rice without *M. oryzae* infection were determined in greenhouse and field conditions. As shown in Fig. 2, the growth parameters of E+M– rice were significantly higher than those of E–M– rice at 28 days posttreatment (dpt) (Fig. 2A and B). The production of E+M– rice was also remarkably improved at the yellow ripening stage with the thousand-grain weight (TGW) significantly increased by 2.1% (Fig. 2C and D). In the field experiment, the agronomic traits of E+M– rice were also significantly better than that of E–M– rice, with the TGW markedly increased by 2.2% (Fig. 2E and F).

The inoculation of OsiSh-2 was also confirmed. In the greenhouse, the reisolation frequency of OsiSh-2 from inner tissues of E+M– rice seedling was 50.0%, while no OsiSh-2 was isolated from the E–M– rice (see Fig. S1A in the supplemental material). The relative OsiSh-2 biomass in rice, indicated by the ratio of *ShRpoA* gene (a DNA-directed RNA polymerase subunit alpha of OsiSh-2) versus *OsUbq* gene, was 74.9-fold higher than that in E–M– rice (see Fig. S1B). In the field samples, the reisolation frequencies of OsiSh-2 from E+M– and E–M– rice seedling were 53.8% and zero, respectively (see Fig. S1A).

10.1128/mBio.01566-21.1 FIG S1 OsiSh-2 can stably colonize in rice tissues. (A) Reisolation rate (%) of OsiSh-2 from rice tissues grown in the greenhouse or field conditions. NF, not found. (B) The relative growth of OsiSh-2 was calculated using the threshold cycle value (*C_T_*) of *ShRpoA* DNA (an RNA polymerase subunit alpha gene of OsiSh-2) versus the *C_T_* of *OsUbq* DNA (a genomic ubiquitin gene of rice) by DNA-based qRT-PCR in rice leaves. Values are means ± the SD (*n* = 3). E–M–, rice foliar presprayed with water; E+M–, rice foliar presprayed with OsiSh-2 spore suspension (10^8^ spores ml^−1^) without M. oryzae infection. Download FIG S1, TIF file, 0.4 MB. Copyright © 2021 Gao et al. 2021 Gao et al. https://creativecommons.org/licenses/by/4.0/ This content is distributed under the terms of the Creative Commons Attribution 4.0 International license.

For plants, the defense and growth pathways are intertwined and conflicted. To cope with the trade-off between growth and defense, plants developed symbiotic relationships with microbes. Some rhizobacteria were reported with the capacity of plant growth stimulation and rice blast resistance induction in greenhouse conditions. However, the experiments in the laboratory do not necessarily reflect the potential of microbes under variable field conditions. Among the very limited reports about the field performances, the recent examples including the rhizobacteria *Bacillus* spp. (34) and Cladosporium cladosporioides C24G (35) showed the capability of significantly reducing rice blast disease severity with increased grain yield during 2-year field experiments. In this study, our consecutive greenhouse and field trials revealed that rice endophytic OsiSh-2 also has positive effects on rice growth as well as disease resistance. Endophytic actinobacteria might represent a novel element in coping with the trade-off between growth and defense in the host plants. We thus used OsiSh-2-rice symbiont to study the involved mechanisms of this balance modification of the growth and defense.

### Endophytic OsiSh-2 establishes a flexible interaction with host rice and the beneficial effects on rice is an active process.

Although OsiSh-2 is a beneficial microbe of rice, it also might be recognized as an alien organism by rice and need confront the rice defense system to colonize (24). On the other hand, the plant immune system is tuned, and the activation of immune response is costly, which might lessen plant fitness (36). Thus, the level of immune activation should be carefully regulated between OsiSh-2 and rice.

In the greenhouse test without pathogen stress, the growth of E+M– rice was impaired compared to the E–M– rice at 7 dpt by OsiSh-2. However, E+M– and E–M– rice showed a comparable phenomenon at 14 dpt, and E+M– rice possessed a more flourishing growth than E–M– rice at 21 dpt (see Fig. S2). This result indicated that the initial defects in plant growth in OsiSh-2-infected plants are overcome and reversed in later growth stages.

10.1128/mBio.01566-21.2 FIG S2 Initial defects in plant growth in OsiSh-2 treated rice are overcome and reverses in later growth stages. (A) Representative photographs of rice at 7, 14, and 21 days posttreatment (dpt) by OsiSh-2 foliar spraying at a concentration of 10^8^ spores ml^−1^. The images are separately shown as the aboveground parts and roots of rice. (B) The seedling growth traits are presented by the height (cm) of above-ground seedlings, root length (cm), and fresh weight of the complete plants at 21 dpt. Values are means ± the SD (*n* = 50). Bars with asterisks are significantly different, as determined by the Tukey-Kramer test (*, *P* < 0.05; **, *P* < 0.01; ***, *P* < 0.001). The percent changes followed by “+” or “–” above the bars were calculated by using the formula: percentage change = [(value of treated rice) – (value of untreated rice)]/(value of untreated rice) × 100%. E–M–, rice foliar presprayed with water; E+M–, rice foliar presprayed with OsiSh-2 spore suspension (10^8^ spores ml^−1^) without Magnaporthe oryzae infection. Download FIG S2, TIF file, 2.7 MB. Copyright © 2021 Gao et al. 2021 Gao et al. https://creativecommons.org/licenses/by/4.0/ This content is distributed under the terms of the Creative Commons Attribution 4.0 International license.

**FIG 3:**
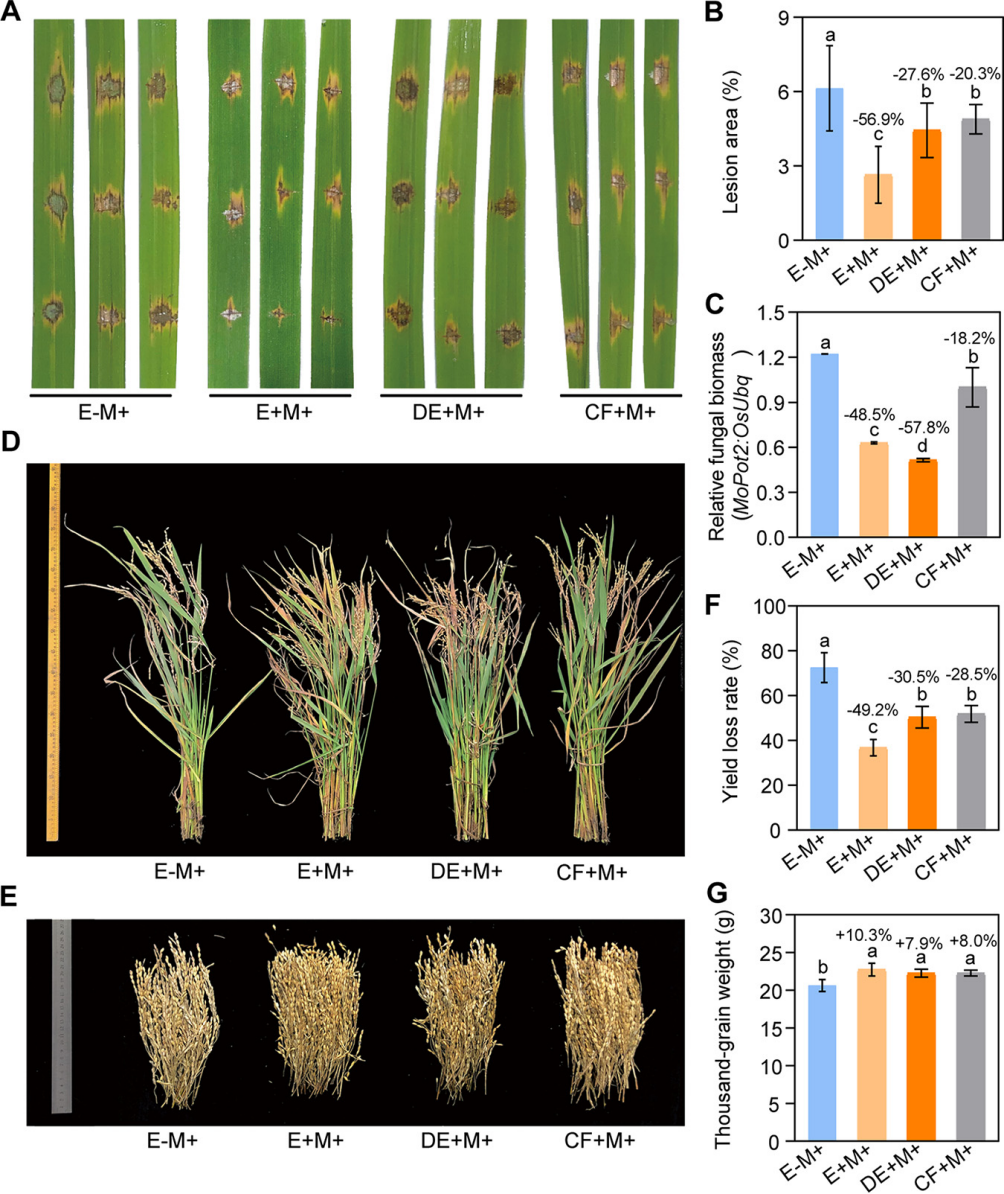
Various active forms of *S. hygroscopicus* OsiSh-2 enhance rice resistance against the rice blast pathogen. (A) Images of blast lesions on detached rice leaf segments 5 days after punch inoculation of *M. oryzae* at a concentration of 10^5^ conidia ml^−1^. Values are means ± the SD (*n* > 20). (B) The relative lesion area of detached rice leaves was measured by using ImageJ. Values are means ± the SD (*n* > 10). (C) The relative fungal growth of *M. oryzae* was calculated using the threshold cycle value (*C_T_*) of *MoPot2* DNA (an inverted repeat transposon of *M. oryzae*) versus the *C_T_* of *OsUbq* DNA (a rice genomic ubiquitin gene) by DNA-based qRT-PCR at 7 days postinfection by pathogenic *M. oryzae* in rice leaves. Values are means ± the SD (*n* = 3). (D) Representative photograph of 4-month-old E–M+, E+M+, DE+M+, and CF+M+ rice after the panicle blast in the year 2020. (E) Representative photograph of all panicles in five infected rice plants. (F and G) Yield loss rate (%) and thousand-grain weight (g) of 4-month-old rice after the panicle blast in the year 2020. Values are means ± the SD (from 20 independent rice plants). Bars with different letters are significantly different (ANOVA, *P* < 0.05) according to Duncan’s multiple-range test. The percent changes followed by “+” or “–” above the bars were calculated by using the following formula: percentage change = [(value of treated rice) – (value of untreated rice)]/(value of untreated rice) × 100%.

It is also reasonable to assume that the benefits of OsiSh-2 in host rice are related to a dynamic regulated process and rely on its active form. To test this hypothesis, the effects of OsiSh-2 spores (E+M+), dead OsiSh-2 spores (DE+M+), and cell-free culture filtrate (CF+M+) on rice after *M. oryzae* infection were examined. E–M+ rice was used as a blank control. The detached rice leaves were collected and inoculated with blast by using a punch method. As shown in Fig. 3A to C, the lesions of E+M+, DE+M+, and CF+M+ rice were all significantly smaller than those of the E–M+ rice, and the relative fungal biomass, as indicated by the ratio of *MoPot2* gene (an inverted repeat transposon of *M. oryzae*) to the *OsUbq* gene (a rice genomic ubiquitin gene) in treated rice, was correspondingly decreased. E+M+ rice, in particular, developed a 56.9% smaller lesion area at the wound inoculation sites, with a 48.5% reduction in fungal biomass (Fig. 3B and C). When we replanted the rice plants in the field with blast disease occurrence, the growing status and decreases in yield loss rates for three OsiSh-2-treated rice were still better than for E–M+ rice (Fig. 3D to F). Similarly, the yield of E+M+ rice was much better than other compared groups, with the lowest yield loss rate 49.2% (Fig. 3F) and the highest TGW of 22.7 g (Fig. 3G).

We also investigated the growth of the rice treated as described above without pathogen stress. Similarly, all of the growth parameters of E+M– rice were best in the greenhouse test (see Fig. S3). In the field condition, the best agronomic traits were found in E+ rice, while those of DE+M– and CF+M– rice did not surpass those of E–M– rice (Fig. 2E and F). For example, the TGW of DE+M– rice was 1.8% lower than that of E+M– rice and only 0.4% higher than that of E–M– rice. The TGW of CF+M– rice was even 0.5% lower than that of E–M– rice.

10.1128/mBio.01566-21.3 FIG S3 Streptomyces hygroscopicus OsiSh-2 promotes rice growth depending on its active form. (A) Representative photograph of 24-day-old rice plants treated by water (E–M–), OsiSh-2 spore (E+M–), dead OsiSh-2 spore (DE+M–), and cell-free culture filtrate (CF+M–) without M. oryzae infection under greenhouse conditions. (B) The seedling growth traits are presented as the height (cm) of above-ground seedlings, the dry weight (g) of the complete plants, and the net photosynthetic rate (μmol m^−2^ s^−1^). Values are means ± the SD (*n* = 50). Bars with different letters are significantly different (ANOVA, *P* < 0.05) according to Duncan’s multiple-range test. The percent changes followed by “+” or “–” above the bars were calculated by using the following formula: percentage change = [(value of treated rice) – (value of untreated rice)]/(value of untreated rice) × 100%. Download FIG S3, TIF file, 1.8 MB. Copyright © 2021 Gao et al. 2021 Gao et al. https://creativecommons.org/licenses/by/4.0/ This content is distributed under the terms of the Creative Commons Attribution 4.0 International license.

In short, the active OsiSh-2 exhibited a greater advantage in the enhancement of both growth promotion and defense resistance than did its dead cells and metabolites. This might benefit from the symbiosis relationship between OsiSh-2 and host rice, by which the dynamic accommodation and adaption would happen in facing the various environmental changes. That is, OsiSh-2 might participate in the balance modification of the growth and defense in host rice.

### Cytological, physiological, and genetic transcription characterization confirm the promotion of rice growth and defense in the presence of OsiSh-2.

**FIG 4:**
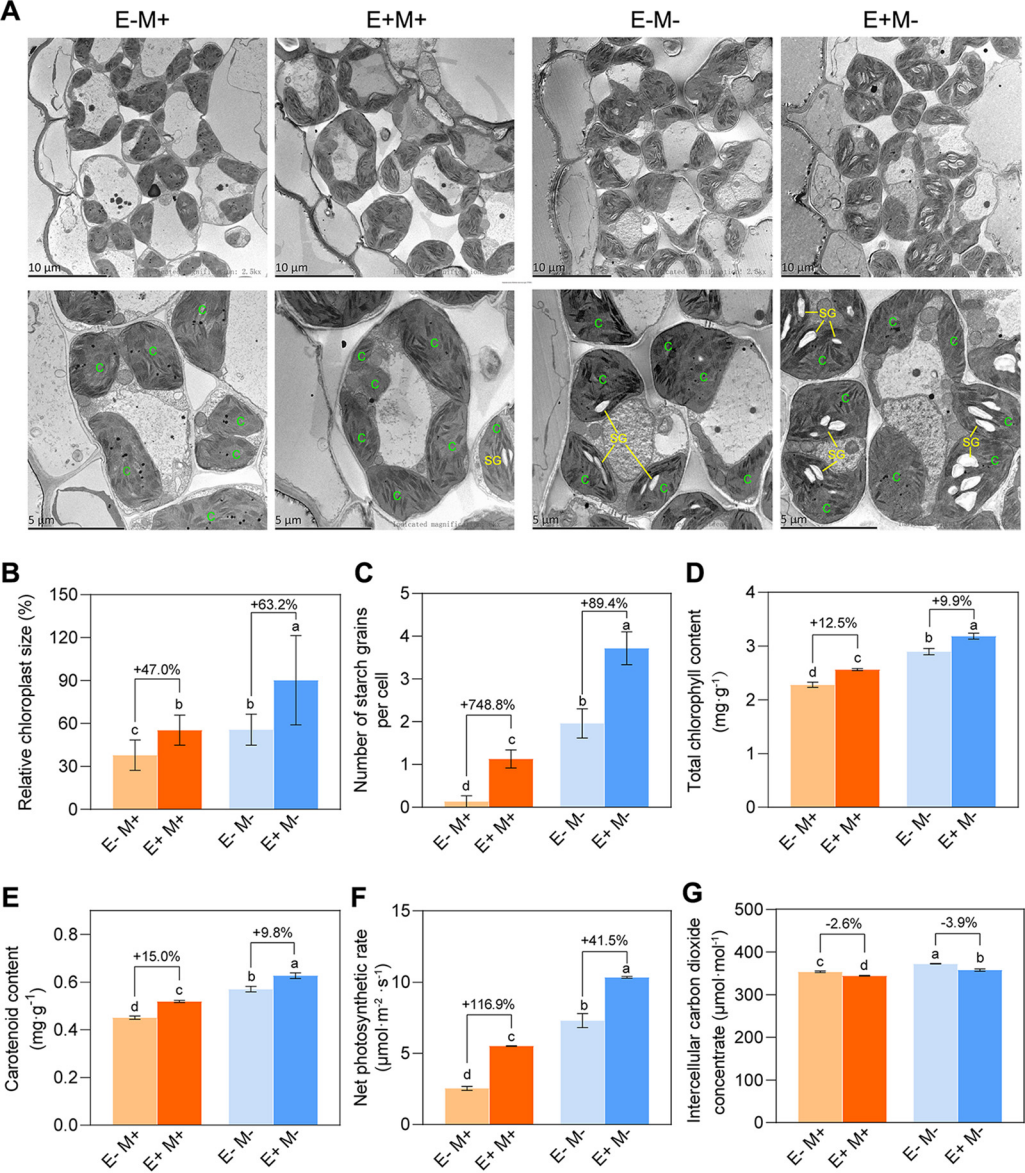
*S. hygroscopicus* OsiSh-2 improves chloroplast development and photosynthesis rate in rice. (A) The internal ultrastructure of leaf cells in the E– and E+ rice at 48 h after M– or M+ treatment was observed by using transmission electron microscopy. Labels: C, chloroplast; SG, starch grain. (B and C) The percentage of relative chloroplast size and the number of starch grains per cell were calculated by using Photoshop CS6 and ImageJ based on counting the numbers of chloroplast pixels and starch grains from 10 to 15 rice cells. (D and E) The chlorophyll contents, including total chlorophyll and carotenoid in E– and E+ rice at 168 h after M– or M+ treatment, were measured by using spectrophotometry. (F and G) The photosynthesis efficiency analysis, including the net photosynthetic rate and intercellular carbon dioxide concentrate in rice samples collected as described for panel C, was determined by using portable photosynthesis system. Values are means ± the SD (*n* = 20). Bars with different letters are significantly different (ANOVA, *P* < 0.05) according to Duncan’s multiple-range test. The percent changes followed by “+” or “–” above the bars were calculated by using the following formula: percentage change = [(value of treated rice) – (value of untreated rice)]/(value of untreated rice) × 100%.

The growth-promoting characters of OsiSh-2 were then confirmed at the subcellular level by transmission electron microscope (TEM) observation of the rice leaves. As shown in Fig. 4A, E+ rice showed a better development of chloroplasts compared to E– rice in both M+ and M– situations, with the relative chloroplast sizes increased by 47.0 and 63.2% (Fig. 4B) and the numbers of starch grains per cell increased by 748.8 and 89.4%, respectively (Fig. 4C). The chloroplast is the organelle where photosynthesis happens and sunlight energy is converted and stored for plant growth and development. As expected, this morphological change was correlated with functional improvement: the growth parameters involved with chloroplasts, including the chlorophyll content and net photosynthetic rate of E+ rice, were all significantly enhanced compared to those in E– rice (Fig. 4D to G).

It should be noted that *M. oryzae* infection led to obvious damage in the chloroplast structure of E–M+ rice leaves, evident as grana revealing an obscure boundary in the laminar system. However, the grana in E+M+ rice still maintained sharply defined areas (Fig. 4A), indicating a protective effect on chloroplast integrity by OsiSh-2. As we knew, in addition to the function of photoautotrophy, chloroplasts also play crucial roles in the activation of defense responses (37). The biosynthesis of defense-related molecules such as salicylic acid and jasmonic acid, as well as secondary messengers, including calcium and reactive oxygen species (ROS), all take place in the chloroplast (38). A recent study showed that a light-harvesting complex II protein LHCB5 in the chloroplast is subject to specially enhance the resistance to *M. oryzae* by the accumulation of ROS. Conversely, the successful colonization and invasion of *M. oryzae* in rice tissues are always accompanied by the destruction of chloroplasts in rice plants (39). Consistent with these reports, our data suggested that OsiSh-2 could not only promote host rice growth by effectively improving the rice chloroplast development but also help the host to enhance disease resistance by alleviating the damaging effects on chloroplast-caused pathogen infection.

In addition to cytological management, plants can develop induced resistance in response to various types of stress stimulation, including pathogen infection or specific beneficial microbes’ colonization (40, 41). Thus, the ROS signaling, which is involved in the immune response, was determined. Spraying OsiSh-2 on the rice leaves led to a quick accumulation of H_2_O_2_ at 0.5 h posttreatment (hpt) in the veins of E+M+ rice leaves, whereas no H_2_O_2_ accumulation was detected in E–M+ rice (see Fig. S4A and B). As a result, the downstream immune responses of H_2_O_2_ messenger, such as callose deposition, which works as an effective barrier in the plant cell wall for blocking the pathogen invasion (42), was induced. By 12 hpt, the number of callose deposition around the stomata was noticeably increased in E+M+ rice leaves, whereas no callose deposition was observed in E–M+ rice (see Fig. S4C and D).

10.1128/mBio.01566-21.4 FIG S4 H_2_O_2_ accumulation and callose deposition are induced by OsiSh-2 treatment in rice leaves. (A) Stereomicroscope observation of H_2_O_2_ accumulation in untreated (E–M–) and OsiSh-2-treated (E+M–) rice at indicated hour posttreatment (hpt) without M. oryzae infection. (B) The abundance of H_2_O_2_ accumulation is represented by the percentage of relative DAB intensities in the same sample as for panel A, which were calculated by Photoshop CS6 and ImageJ based on counting the numbers of DAB staining pixels. (C) Fluorescent microscope observation of callose depositions in the same samples as in panel A. (D) The abundances of callose intensities are represented by the percentage of relative callose intensities in same samples as in panel A) which were calculated by using Photoshop CS6 and ImageJ based on counting the numbers of callose staining pixels. At least 10 typical photographs were selected from 15 randomly selected seedlings in each treatment. Values are means ± the SD, and the experiment was repeated twice. Bars with different letters are significantly different (ANOVA, *P < *0.05) according to Duncan’s multiple-range test. Yellow arrows indicate the callose deposition, mainly around the stomata. Scale bars: 0.5 cm (A), 15 μm (B). Download FIG S4, TIF file, 2.5 MB. Copyright © 2021 Gao et al. 2021 Gao et al. https://creativecommons.org/licenses/by/4.0/ This content is distributed under the terms of the Creative Commons Attribution 4.0 International license.

**FIG 5:**
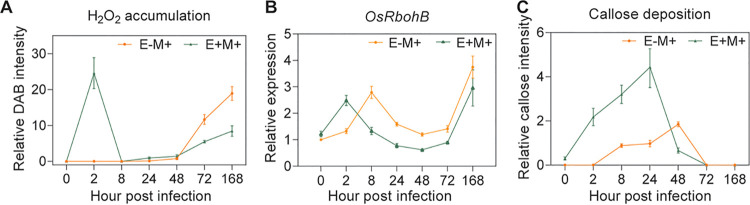
*S. hygroscopicus* OsiSh-2 triggers a defense priming in rice leaves. (A) The abundance of H_2_O_2_ accumulation is represented as the percentage of relative DAB intensities in E+M+ rice leaves at indicated hour postinfection by *M. oryzae*, which were calculated by using Photoshop CS6 and ImageJ based on counting the numbers of DAB staining pixels. (B) The transcriptional levels of the encoding genes for NADPH oxidase (*OsRbohB*) in the same samples as described for panel A were analyzed using qRT-PCR. (C) The abundance of callose intensities are presented as the percentage of relative callose intensities in same samples as described for panel A, which were calculated by using Photoshop CS6 and ImageJ based on counting the numbers of callose staining pixels. The data at 0 h postinfection present the respective levels of H_2_O_2_ accumulation, *OsRbohB* transcription, and callose deposition in E–M– rice. At least 10 typical photographs were selected from 15 randomly selected seedlings in each treatment. Values are means ± the SD, and the experiment was repeated twice.

We next investigated the rice responsive processes during pathogen infection. *M. oryzae* infection led to significant ROS production in rice. Interestingly, this response in E+M+ rice was faster and stronger than that in E–M+ rice. A large amount of H_2_O_2_ accumulated in the E+M+ rice leaves at 1 to 2 h postinfection (hpi) by *M. oryzae*, while the H_2_O_2_ in E–M+ rice just began to accumulate at 24 to 36 hpi (Fig. 5A; see also Fig. S5A). Consistently, the expression of *OsRbohB*, an NADPH oxidase gene involved in deliberating generation of ROS in plants during defense response (43), reached its first peak at 2 hpi in E+M+ rice, which was 10 h earlier than that in E–M+ rice (Fig. 5B). The callose deposition also happened at least 4 h before that in E–M+ rice, and the amount was also dramatically improved in E+M+ rice during 1 to 24 hpi (Fig. 5C; see also Fig. S5B). These results, especially the callose data, presented a typical defense priming response pattern in OsiSh-2-rice symbiont, indicating that OsiSh-2 could raise an immune response in rice by priming (26).

10.1128/mBio.01566-21.5 FIG S5 H_2_O_2_ accumulation and callose deposition are induced in rice leaves after Magnaporthe oryzae infection. (A and B) Stereomicroscope observation of H_2_O_2_ accumulation and fluorescent microscope observation of callose depositions in untreated (E–M+) and OsiSh-2-treated (E+M+) rice at indicated hour post infection (hpi) by *M. oryzae*. The pictures at 0 hpi show the status of H_2_O_2_ accumulation and callose deposition in E–M– rice. The experiment was repeated twice. Scale bars: 200 μm (A); 100 μm (B). Download FIG S5, TIF file, 2.6 MB. Copyright © 2021 Gao et al. 2021 Gao et al. https://creativecommons.org/licenses/by/4.0/ This content is distributed under the terms of the Creative Commons Attribution 4.0 International license.

**FIG 6:**
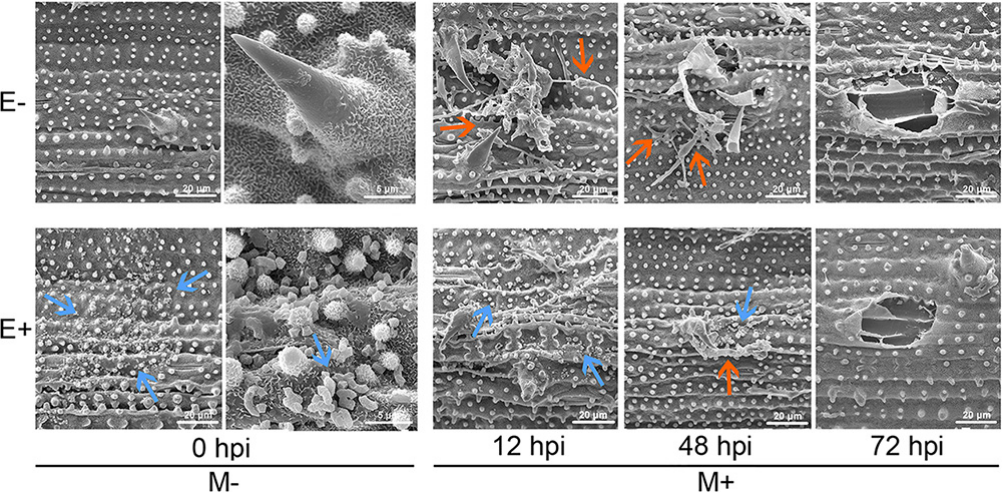
OsiSh-2 colonization of rice is associated with less fungal conidia and host damage. The surface of rice leaves at the indicated hour postinfection (hpi) by *M. oryzae* was observed using a scanning electron microscope. Blue arrows indicate the spores of OsiSh-2, and orange arrows indicate the mycelia or conidia of *M. oryzae*.

Except inducing priming, we thought OsiSh-2 might also have contributed the first line of defense against the pathogen on host rice. In our previous study, both OsiSh-2 and its culture filtrate can strongly hinder the appressorial formation and inhibit the conidium germination of *M. oryzae in vitro*, two main steps for the foliar pathogen *M. oryzae* to infect the rice leaf (33). Correspondingly, the scanning electron microscope observation revealed that *M. oryzae* infection seriously destroyed the surface structures of E–M+ rice leaves. However, the inoculation of OsiSh-2 prior to the blast treatment led to a decreased distribution of *M. oryzae* conidia, and the damaging effects by pathogen infection were alleviated. We even could not observe the mycelia of *M. oryzae* by the first 12 hpi (Fig. 6). The genome annotations revealed that among the 74 secondary metabolites-synthesize gene clusters in actinobacteria OsiSh-2, 67 clusters were involved with the biosynthesis of antibiotic substance, including polyketide synthase and nonribosomal peptide synthetase. We have confirmed the capacity of OsiSh-2 on producing antimicrobial enzymes, plant hormones, antibiotics and siderophores (26–28). These compounds might directly or indirectly inhibit the pathogen, thus decreasing additional transcription and translation costs of the defense-related gene of plants. As a consequence, the saved energy could drive the growth of the host. To elucidate this direct protection of OsiSh-2 on rice, further study of the isolation and purification of the antagonistic products of OsiSh-2 is under way.

### OsiSh-2 dynamically balanced the expression of host proteins involved in defense and growth activity.

**FIG 7:**
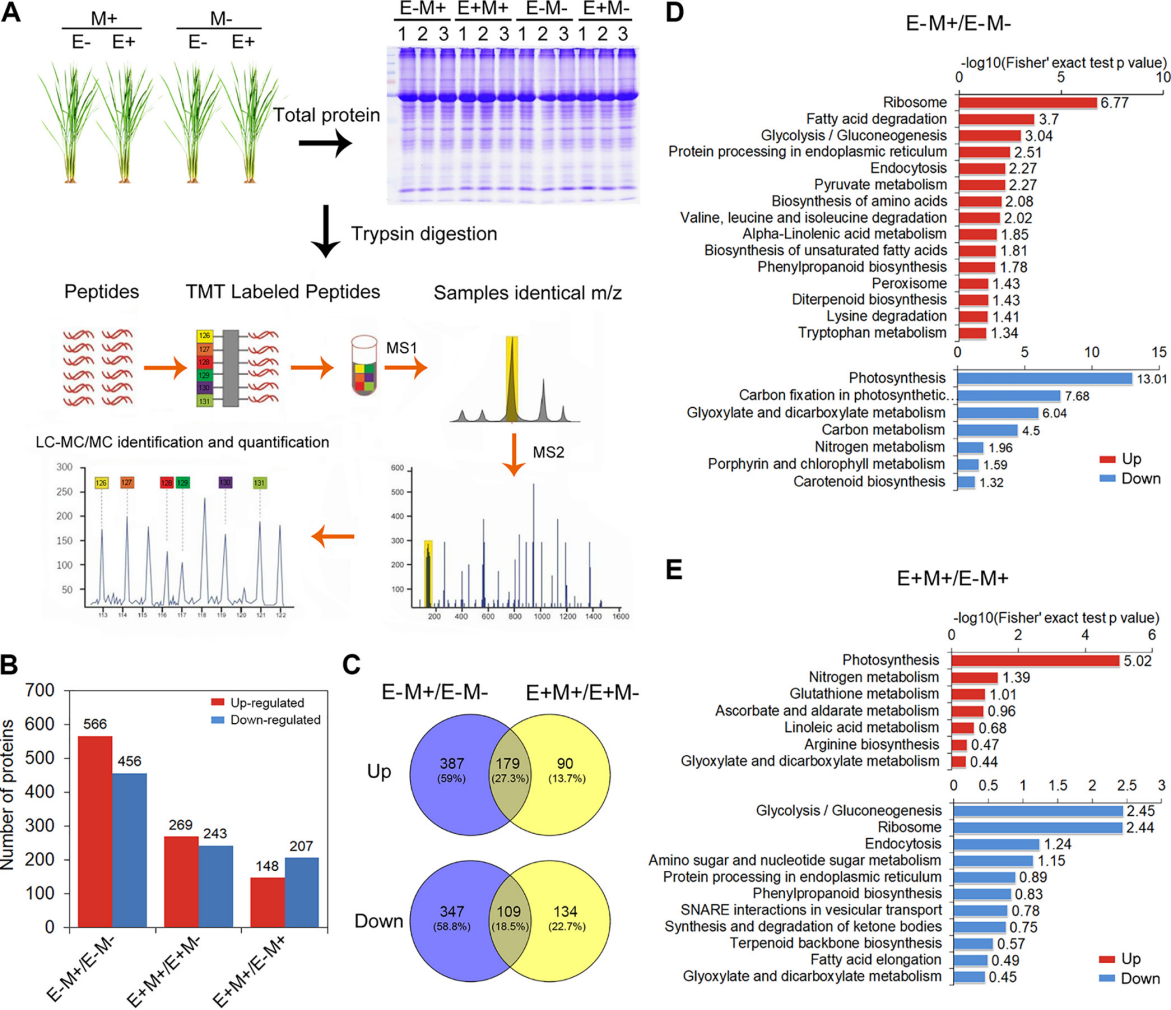
Rice proteomic profiling responds to the treatment of *M. oryzae*. (A) Work flow for comparative proteomics analysis in E– and E+ rice at 48 h after M– or M+ infection. (B) Numbers of up- or downregulated differently expressed proteins (DEPs) in each compared group. (C) The overlap of DEP abundance in the two compared groups (E–M+/E–M– and E+M+/E+M–) is presented in a Venn diagram. (D and E) Up- or downregulated DEPs in the E–M+/E–M– and E+M+/E–M+ groups are presented in heat maps based on an analysis of KEGG pathway enrichment. Membership values are color coded, with red denoting high expression levels and blue denoting low expression levels of proteins.

To gain a deeper understanding of the active OsiSh-2 in the modification of the growth and defense in host rice from the molecular level, the proteomic profiles of E–M+ and E+M+ rice at 48 hpi after *M. oryzae* infection (about 9 dpt by OsiSh-2 or water control) were identified by using mass spectrometry-based TMT labeling quantitative proteomics analysis. The mass spectrometry proteomics data are available via the ProteomeXchange Consortium with the data set identifier PXD026348. The workflow is shown in Fig. 7A. Altogether, 6,392 proteins were identified and 5,163 proteins were quantified (see Fig. S6A). The numbers of differently expressed proteins (DEPs) in each compared group are shown in Fig. 7B and C. The identities of DEPs and the magnitude of their changes are listed in Table S1. Volcano plots show the quality of the identified peptides and the change levels of the DEPs that had significantly altered in each group, respectively (see Fig. S6B and C).

10.1128/mBio.01566-21.6 FIG S6 Overview of the quantitative proteomics analysis. (A) The number of spectra, peptides, proteins, etc., identified using LC/MS-MS proteomics by searching against Oryza sativa subsp. *japonica* (rice) proteome database (UP000059680) at a critical false discovery rate (FDR) of 1% are shown in the column diagram. (B) The score of identified peptides is shown in a volcano plot with red dots. (C) The differentially expressed proteins are visualized by volcano plot. Downregulated proteins are indicated by blue dots, while upregulated proteins are indicated in red. Those that are not significantly altered are shown in gray in the center. Download FIG S6, TIF file, 1.1 MB. Copyright © 2021 Gao et al. 2021 Gao et al. https://creativecommons.org/licenses/by/4.0/ This content is distributed under the terms of the Creative Commons Attribution 4.0 International license.

10.1128/mBio.01566-21.8 TABLE S1 Identities of DEPs and the magnitude of their changes in each compared group. Download Table S1, XLSX file, 0.5 MB. Copyright © 2021 Gao et al. 2021 Gao et al. https://creativecommons.org/licenses/by/4.0/ This content is distributed under the terms of the Creative Commons Attribution 4.0 International license.

Compared to the *M. oryzae* uninfected control, 1,022 DEPs were found in the *M. oryzae*-infected E– rice (E–M+/E–M–). However, when pretreated with OsiSh-2 (E+M+/E+M–), the number of DEPs remarkably decreased to 512. These data indicated that compared to the OsiSh-2-pretreated rice, nontreated rice might induce a more extensive process in response to *M. oryzae* infection. Recently, it was considered that desensitizing the stress responses could be a more efficient strategy to achieve both stress resistance and growth (44). Such an idea matches our results. From the perspective of energy metabolism, this result indicated that E+M+ rice was in a more homeostatic state compared to E–M+ rice when facing the pathogen stress. Taking the function-annotated DEPs into consideration, some proteins likely regulating the defense response, energy metabolism, and nutrient metabolism were analyzed emphatically.

There are 566 upregulated DEPs in E– rice after *M. oryzae* infection (E–M+/E–M–) (Fig. 7B). KEGG analysis showed that these proteins are mainly involved with energy-supply processes, such as oxidoreductase (3.8-fold) in fatty acid degradation and pyruvate kinase (2.1-fold) in glycolysis/gluconeogenesis, and energy-consuming processes, such as 30S ribosomal protein S4 (1.8-fold) in ribosome, asparagine synthetase (7.1-fold) in amino acid metabolism, putative dynamin homolog (1.8-fold) in endocytosis, and stemod-13(17)-ene synthase (4.5-fold) in diterpenoid biosynthesis (Fig. 7D). This result indicated that the pathogen stress induced the defense responses, which might be correlated with an energy consumption. This was in line with the previous reports that the disease resistance is an energy-consuming process, leading to the growth-defense trade-off in plants (4, 9, 10, 13). Correspondingly, we found that the 456 downregulated DEPs in E–M+ rice were mainly related to the energy fixation and nutrient absorption pathways, such as ferredoxin (0.4-fold) and ATP synthase epsilon chain (0.3-fold) in photosynthesis, phosphoenolpyruvate carboxylase (0.5-fold) and ribulose bisphosphate carboxylase large chain (0.5-fold) in carbon metabolism, and glutamine synthetase (0.6-fold) in nitrogen metabolism (Fig. 7B and D).

However, the colonization of OsiSh-2 in rice mitigated the overconsumption of energy caused by *M. oryzae*. Among induced proteins in *M. oryzae-*infected rice described above, most DEPs (*n* = 387) were not activated when OsiSh-2 was inoculated in E+M+ rice (Fig. 7C). These findings indicated again the role of OsiSh-2 in maintaining a relative homeostasis in the stress response. For instance, ribosome assembly is the most energy-demanding process linked to cell growth and defense response due to its requirement for coordinated production of processed ribosomal RNAs, proteins, and biogenesis factors (45). A total of 25 ribosomal proteins significantly accumulated in E–M+ rice (upregulated 1.3- to 1.8-fold), while they were not activated expression in E+M+ rice (see Table S2A). Endocytosis is also an energy-consuming process by which cells internalize substances from their external environment. There were also 17 endocytosis proteins (1.3- to 1.8-fold) only activated in E–M+ rice (see Table S2A). These results indicated that during the battle with *M. oryzae*, the presence of OsiSh-2 led to less activation of energy-consuming pathways than in E–M+ rice. The saved energy might be shifted to maintain normal rice growth. Correspondingly, we found that more than half of downregulated DEPs (*n* = 347) in E–M+ rice did not exhibit a reduced expression level in E+M+ rice (Fig. 7C). There were 15 proteins in the photosynthesis process, 31 proteins in the carbon metabolism pathway, and 6 proteins involved in nitrogen metabolism, which all decreased the expression by 0.5- to 0.7-fold in E–M+ rice (see Table S2B).

10.1128/mBio.01566-21.9 TABLE S2 Number of up- or downregulated DEPs in different compared groups. Download Table S2, DOC file, 0.10 MB. Copyright © 2021 Gao et al. 2021 Gao et al. https://creativecommons.org/licenses/by/4.0/ This content is distributed under the terms of the Creative Commons Attribution 4.0 International license.

To be clearer, we then compared the expression levels of DEPs between E+M+ and E–M+ rice. The upregulated pathways in E+M+/E–M+ group were enriched in photosynthesis and nitrogen metabolism, e.g., the ATP synthase epsilon chain (2.6-fold) and ferredoxin (2.5-fold) in photosynthesis and glutamine synthetase (1.4-fold) in nitrogen metabolism (Fig. 7E). On the other hand, the downregulated pathways in the E+M+/E–M+ group were mainly related to energy supply or energy-consuming processes, such as alcohol dehydrogenase 1 (0.7-fold) in glycolysis/gluconeogenesis, 40S ribosomal protein S30 (0.5-fold) in ribosome, putative dynamin homolog (0.6-fold) in endocytosis, and protein disulfide isomerase-like 1-5 (0.6-fold) in protein processing in the endoplasmic reticulum (Fig. 7E). Combined with the results presented above from field and greenhouse trials, these results indicated that OsiSh-2 pretreatment might positively modulate the expression profile of host rice to optimize the allocation of resources between growth and defense.

Interestingly, we found that the expression levels of proteins involved in the glutathione and ascorbate/aldarate metabolism were significantly higher in E+M+ rice than in E–M+ rice. These metabolic processes play crucial roles in maintaining cellular redox homeostasis and detoxifying in living organisms (46). In addition, three peroxidases involved in oxidative stress response showed 1.3- to 2.0-fold overproduction only in E+M+ rice. Peroxidases participate in the release or consumption of ROS, which can repair cell damage caused by ROS and catalyze the formation of lignin in plants (46). In addition, a recent study showed that the modulation of apoplastic ROS homeostasis by a growth-related transcription factor (Homolog of BEE2 Interacting with IBH 1 [HBI1]) can regulate the trade-off between growth and immunity in Arabidopsis thaliana (9). Thus, these results indicated that OsiSh-2 might also be conducive to maintaining the ROS homeostasis in rice during pathogen infection, consequently contributing to the balance between growth and defense in the host.

To verify the proteomic data, the expression levels of the genes encoding 12 proteins involved in the energy-supply or energy-consuming pathways and 6 proteins related to the photosynthesis or nitrogen/carbon metabolism were detected at 48 hpi. In agreement with the proteomic analysis, the evoked levels of eight genes, such as *ADH2* and *PGAM-i* in glycolysis/gluconeogenesis metabolism, *CNX* in the protein processing in endoplasmic reticulum process, *FDH* in the biosynthesis of secondary metabolite, and *RABA2a* in the endocytosis, were much lower in E+M+ rice than in E–M+ rice, when respectively compared to untreated E–M– rice. The transcript abundances of *atpE* and *LFNR2* related to the photosynthesis, *GLN2* and *CAs* in the nitrogen metabolism, and *ALDOA* in the carbon metabolism showed significantly higher levels in E+M+ rice than in E–M+ rice (see Fig. S7). Some genes, such as *Rps4-30* and *RPS17* in the ribosome, were not detected to have the same change tendency as their encoded proteins, likely reflecting their fast turnover inside the cell.

10.1128/mBio.01566-21.7 FIG S7 qRT-PCR verification of the DEPs associated with the defense response and growth regulation. The encoding genes for alcohol dehydrogenase 2 (*ADH2*) and 2,3-bisphosphoglycerate-independent phosphoglycerate mutase (*PGAM-i*) in glycolysis/gluconeogenesis pathway; stemod-13(17)-ene synthase (*KSL11*) in the diterpenoid biosynthesis; 40S ribosomal protein S30 (*Rps4-30*) and 40S ribosomal protein S17 (*RPS17*) in the ribosome; calnexin homolog (*CNX*) in the protein processing in endoplasmic reticulum; 3-ketoacyl-CoA synthase (*FDN*) and probenazole-inducible protein PBZ1 (*PBZ1*) in the biosynthesis of secondary metabolites; leucine aminopeptidase 1 (*LAP1*) in the glutathione metabolism; Os05g0105100 protein (*RABA2a*), ADP-ribosylation factor GTPase-activating protein (*AGD9*), and putative dynamin homolog (*DRP2a*); ATP synthase epsilon chain gene (*atpE*), ferredoxin–NADP reductase (*LFNR2*), and ferredoxin (*FDX3*) in the photosynthesis; glutamine synthetase (GLN2) and carbonic anhydrase (CAs) in nitrogen metabolism; fructose-bisphosphate aldolase (*ALDOA*) in E– and E+ rice leaves at the indicated hour post infection (hpi) by M. oryzae were detected using qRT-PCR. Values are means ± the SD, *n* = 3 (technical repeats). Bars with different letters are significantly different (ANOVA, *P* < 0.05) according to Duncan’s multiple-range test. Download FIG S7, TIF file, 1.2 MB. Copyright © 2021 Gao et al. 2021 Gao et al. https://creativecommons.org/licenses/by/4.0/ This content is distributed under the terms of the Creative Commons Attribution 4.0 International license.

**FIG 8:**
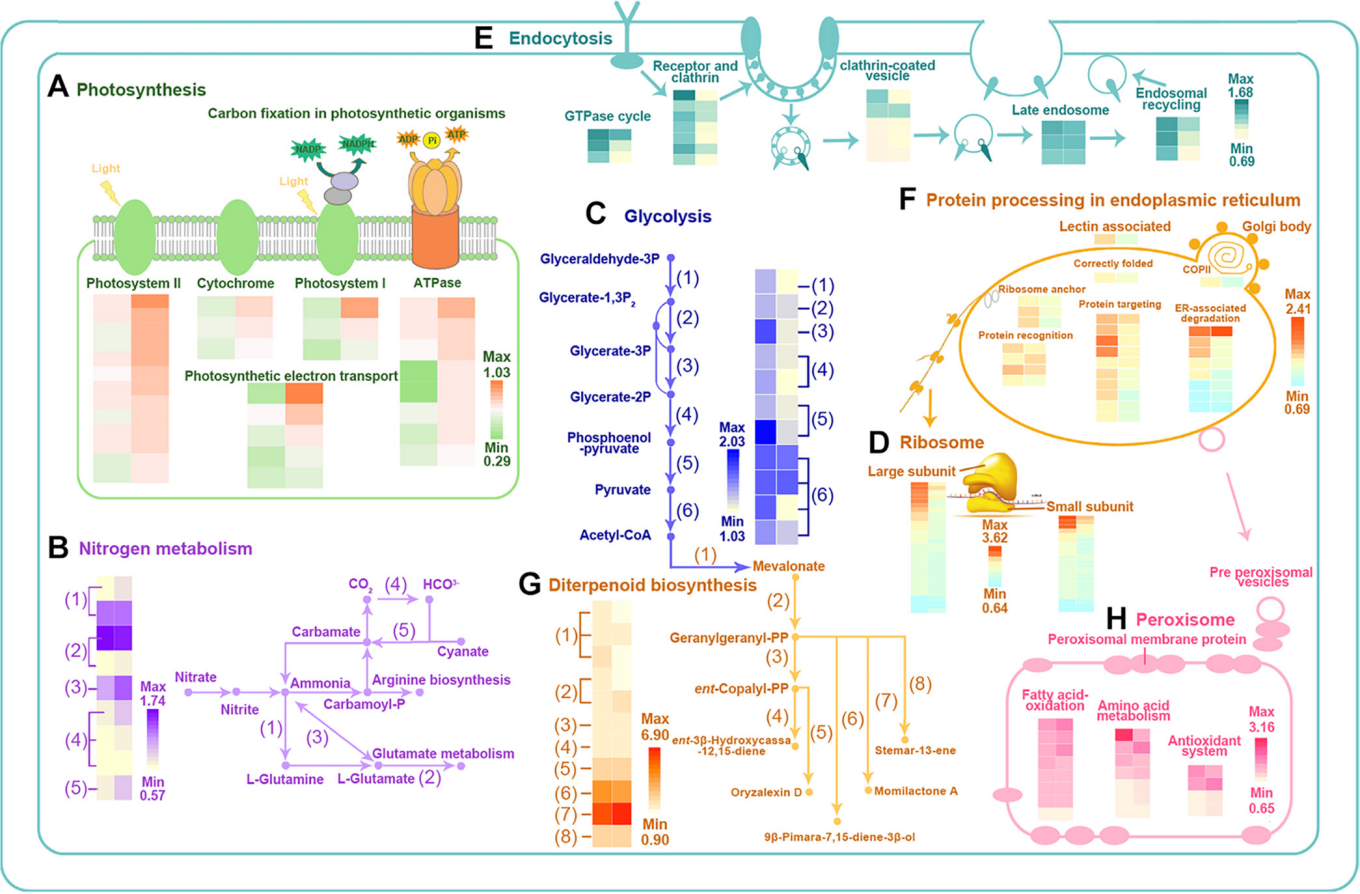
Proteomic features of various cellular processes response to *M. oryzae* in rice. The biological processes significantly varied by OsiSh-2 inoculation in E+ rice from those in E– rice after *M. oryzae* infection sre presented based on the KEGG pathway analysis, including (i) energy-fixation pathways: photosynthesis (A) and nitrogen metabolism (B); (ii) energy-supply pathways: glycolysis (C); and (iii) energy-consuming pathway: ribosome (D), endocytosis (E), protein processing in endoplasmic reticulum (F), diterpenoid (G), and peroxisome (H). Metabolic steps are represented by arrows. The expression levels for the marker proteins in two compared groups (E–M+/E–M– [left] and E+M+/E–M+ [right]) are presented by the heat map.

The protein expression profiles confirmed that pathogen infection led to a balance between growth and defense, but OsiSh-2 mitigates this conflict by attenuating energy-consuming defense-related responses and shifting toward growth activities. As shown in Fig. 8, a schematic regulation model of OsiSh-2 was constructed based on the proteomic data.

### Conclusions.

**FIG 9:**
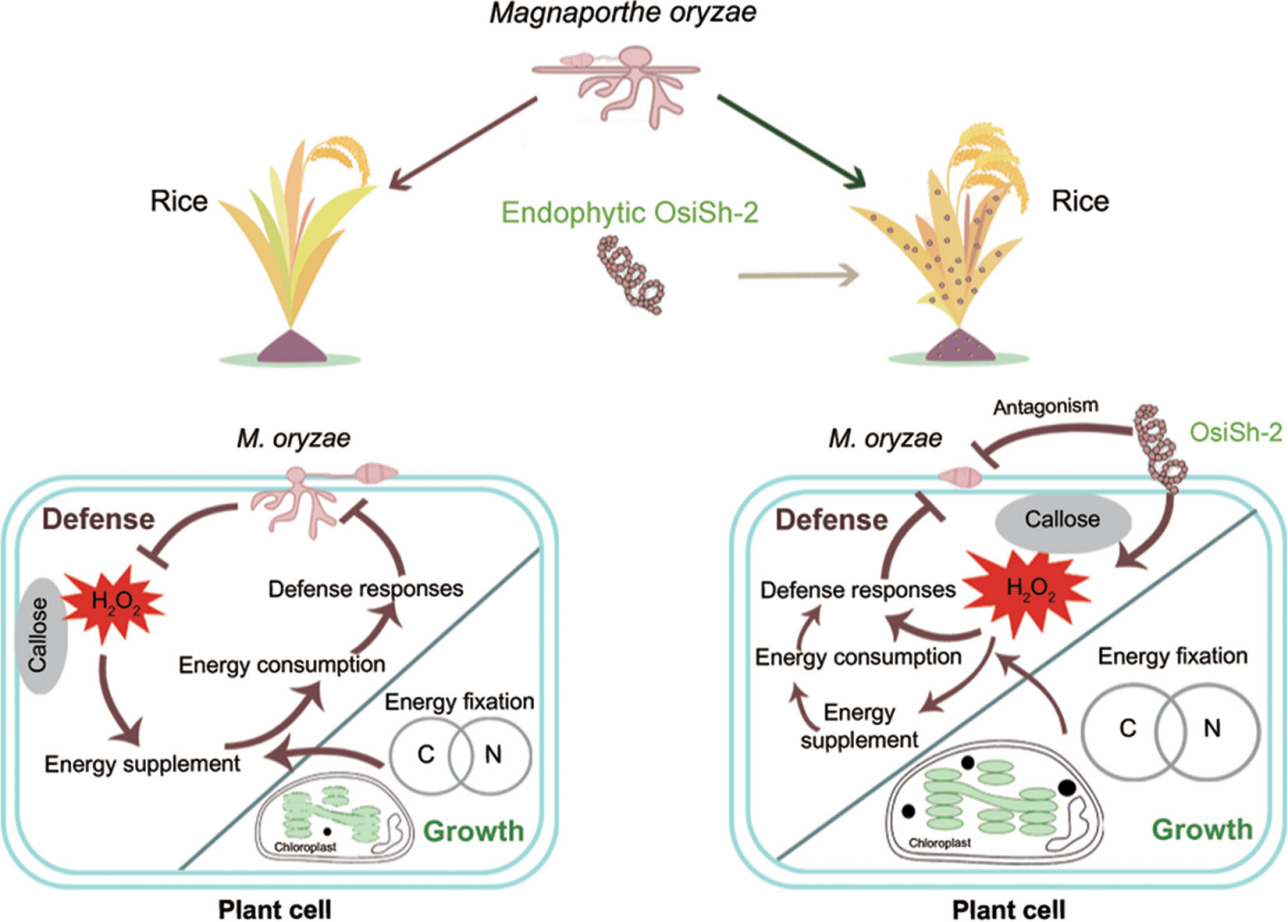
Positive regulatory loop modulating the balance between plant growth and defense against pathogenic *M. oryzae* by endophytic *S. hygroscopicus* OsiSh-2.

We demonstrated the contribution of endophytic actinobacteria OsiSh-2 to promoting both yield and disease resistance in host rice by sustaining a balance between growth and immune defense. We confirmed that the enhanced protection effect of the OsiSh-2 is based on a priming mechanism instead of consuming energy to express many host defense-related genes and proteins. The saved energy is beneficial for plant growth, such as improving photosynthesis activity. A schematic model of OsiSh-2-rice symbiont in responding to blast pathogen *M. oryzae* was constructed (Fig. 9). The ability of OsiSh-2 to achieve both disease resistance and high productivity is likely to increase with a better understanding of the relationship between disease-response and fitness pathways. This sophisticated regulation through a mutual reaction between OsiSh-2 and rice supports the application of endophytic actinobacterial strains as plant inoculants or plant-strengthening agents in agriculture and forestry.

## MATERIALS AND METHODS

### Plant materials and growth conditions.

Rice (Oryza sativa) was cultured in the field at the Yahua Seeds Science Research Institute, Longping High-Tech in Changsha, Hunan, China (YSSRI; 28°11′49′′N, 112°58′42′′E), to assess disease resistance and for yield evaluation. Plants in growth chambers were grown for *M. oryzae* inoculations and immune and comparative proteomic assays. The growth chambers were set as follows: 16/8-h light/dark cycles at 26°C and 80% humidity. The rice cultivar used in the study is Xiang’aizao 7, a cultivar susceptible to *M. oryzae*.

### Endophyte and pathogenic fungus materials and growth conditions.

Endophytic Streptomyces hygroscopicus subsp. *hygroscopicus* OsiSh-2 (GenBank accession number GCA_001705785.1, China General Microbiological Collection Center accession number CGMCC-8716) was isolated from rice sheath and routinely cultured in International Streptomyces Program 2 solid medium at 30°C, as described previously (33). The rice blast pathogenic fungus *M. oryzae* 70-15, which is compatible with Nip, was routinely cultured on solid complete medium at 28°C, as described previously (47). Spore suspensions of OsiSh-2 and 70-15 were prepared as described previously (48).

### Greenhouse tests.

Surface-sterilized rice seeds were sown into pots containing autoclaved soil and grown in a growth chamber. A spore suspension of OsiSh-2 (10^8^ spores ml^−1^) was sprayed on rice leaves at the two-leaf stage. Water containing 0.2% (vol/vol) Tween 20 was used as a control. A conidial suspension of *M. oryzae* (10^5^ conidia ml^−1^) was then sprayed a few days later when the rice plants were at the three-leaf stage. Infected rice plants were placed in darkness overnight at 26°C and then transferred to the growth chamber. The disease index and plant height were determined at 7 days postinfection by *M. oryzae*, as described previously (48). Independent experiments were repeated twice with three replicates and 50 plants per replicate.

### Field tests.

Surface-sterilized seeds were sown in the field in Liuyang, Hunan, China (YSSRI, 28°10′02′′N, 113°38′16′′E). The OsiSh-2 treatment was the same as that done in the greenhouse assays, and spraying commercial fungicide tricyclazole (1.5 μg/ml) containing 0.2% (vol/vol) Tween 20 was added as a positive control (49). The rice seedlings were surrounded by infected seedlings to mimic a natural infection. The disease index and plant height for 1-month-old rice plants after the seedling blasts were determined, as described previously (48). In detail, the disease severity of each seedling blast was evaluated according to the standard evaluation system for rice (International Rice Research Institute, 2013). The disease level was scored on a scale from 0 to 9 as follows: 0, no lesions observed; 1, small brown specks of pin-point size without a sporulating center; 2, larger brown specks but <1 mm in diameter; 3, small (roundish to slightly elongated) necrotic gray spots 1 to 2 mm; 4, typical susceptible blast lesions (spindle-shaped) 3 mm or longer, infecting <4.0% of the leaf area; 5, typical blast lesions infecting 4.1 to 10.0% of the leaf area; 6, typical blast lesions infecting 10.1 to 25.0% of the leaf area; 7, Typical blast lesions infecting 25.1 to 50.0% of the leaf area; 8, typical blast lesions infecting 50.1 to 75.0% of the leaf area; and 9, typical blast lesions infecting >75.1% of the leaf area. The disease index was computed using the following equation: disease index = Σ(number of diseased leaves at all levels × assessed disease level value)/(total number of sampled leaves × highest disease level value) × 100%. The experiment was repeated twice with three replicates per treatment and 100 plants/replicate. The disease index, yield loss rate, and agronomic traits, including the effective tiller number, panicle length, and thousand-grain weight (TGW) for 4-month-old rice after the panicle blast were detected. The panicle with at least five filled grains is defined as effective panicle. The disease index of the panicle blast = Σ(number of panicles at all levels × assessed disease level value)/(total number of sampled panicles × highest disease level value) × 100%. The yield loss rate = Σ [(number of empty grains per panicle/total grains per panicle)]/total number of collected panicles × 100%. Each treatment was carried out in two different fields, and three replicates per treatment in each field using a randomized complete block design. Each replicate contained 100 rice plants for seedling blasts and 20 for panicle blasts.

The field experiment without pathogen stress was conducted in paddy fields located at YSSRI (28°3167″N, 112°6782″E). Approximately 20-day-old rice seedlings were transplanted to the paddy fields, with three repeats for each treatment. The planting density was set as 35 hills/m^2^ (one seedling per hill, 16.5-cm hill spacing, 19.8-cm inter-row spacing), as described previously (50). Data were collected from the 20 plants from the middle two rows. Several agronomic traits, including the number of effective tillers, the panicle length, and the TGW, were measured.

### Punch inoculation analysis.

The basal resistance of rice plants against *M. oryzae* was evaluated by the punch inoculation method, as described previously, with some modifications (51). In brief, detached leaves of rice at the three-leaf stage under the greenhouse conditions were lightly wounded with a mouse ear punch. Then, 10 μl of spore suspension of *M. oryzae* was added to the wound. The inoculated rice leaves were placed in plates with water for restoring humidity, kept in darkness overnight at 26°C, and then transferred to the growth chamber for 5 days. The lengths of the lesions were then measured.

### Fungal and endophytic biomass analysis.

The fungal and endophytic biomass in rice plants was quantified as described previously with a slight modification (51). In brief, 0.05-g rice tissues were collected for DNA extraction using a Dzup (plant) genomic DNA isolation reagent kit (Sangon Biotech, Shanghai, China) according to the manufacturer’s specifications. DNA-based quantitative real-time PCR (qRT-PCR) was performed using a CFX96 real-time system instrument (Bio-Rad, USA). The reactions were conducted using SYBR Premix *Ex Taq* TM II (Tli RnaseH Plus; TaKaRa, Dalian, China) according to the manufacturer’s specifications. The relative fungal growth was calculated using the threshold cycle value (*C_T_*) of *MoPot2* DNA (an inverted repeat transposon of *M. oryzae*) against the *C_T_* of *OsUbq* DNA (a rice genomic *ubiquitin* gene), while the relative OsiSh-2 growth was calculated using the *C_T_* of *ShRpoA* DNA of OsiSh-2 against the *C_T_* of *OsUbq* DNA of rice as a ratio (*ShRpoA*/*OsUbq*), represented by the equation 2*^CT^*^(^*^OsUbq^*^) – ^*^CT^*^(^*^ShRpoA^*^)^, as described in a previous report (51). The primers for DNA-based qRT-PCR are listed in Table S3.

10.1128/mBio.01566-21.10 TABLE S3 RT-qPCR primers used in this study. Download Table S3, DOC file, 0.04 MB. Copyright © 2021 Gao et al. 2021 Gao et al. https://creativecommons.org/licenses/by/4.0/ This content is distributed under the terms of the Creative Commons Attribution 4.0 International license.

### Endophyte reisolation analysis.

For OsiSh-2 reisolation from inner host plants, the rice tissues were excised and subjected to four-step surface sterilization as described previously (52). In brief, the rice tissues were (i) washed in 4% NaClO for 6 min, (ii) washed in absolute ethanol for 30 s, (iii) rinsed in deionized water for 30 s, (iv) washed in 5% Na_2_S_2_O_3_·5H_2_O for 5 min, and (v) finally rinsed in deionized water. After drying under laminar airflow for1 to 3 h, the rice tissues were distributed onto the Mannitol soya flour (MS) medium, a commonly used medium for actinomycetes isolation, and incubated at 27°C for up to 14 days. Benomyl and nalidixic acid were added to the isolation media at 50 mg liter^−1^ to control endophytic fungal and bacterial growth, respectively. The efficiency of the surface sterilization procedure was assessed by adding 200 μl of the final rinse water to the MS medium. Surface sterilization method was considered successful when no microbes grew on the MS plates.

### Microscopy imaging analysis.

For the scanning electron microscopy observation, the leaf samples were fixed and observed as previously described (48). For the transmission electron microscope observation, leaf pieces of approximately 1 to 2 mm^2^ were cut from the middle of leaves and then immediately soaked in a fixation buffer (2.5% glutaraldehyde in 100 mM phosphate buffer [pH 7.4]). Polymerization and staining of the leaf samples were performed as described previously (38). The machine (HT-7700; Hitachi, Japan) was operated at an accelerating voltage of 100 kV. The percentage of relative chloroplast size was determined by counting the total chloroplast intensity in a single cell versus the total intensity of the corresponding cell from 10 to 15 cells of rice using Photoshop CS6 (Adobe Systems) and ImageJ v1.49 (National Institutes of Health), as described previously (48).

### Analysis of the effects of *OsiSh-2* on rice growth and yield.

Under the growth chamber condition, surface-sterilized rice seeds were germinated, and one part was transferred to plates with International Rice Research Institute nutrient solution for morphologic observation and for evaluation of growth parameters, including the height of shoots, the dry weights of whole plants, and the net photosynthetic rate, as described previously (50). Another part was sown into pots containing autoclaved soil for yield assessment. The panicles of 4-month-old rice were collected to determine the panicle length and TGW. The morphologies of rice seedling roots were determined by computerized scanning (STD 1600; Regent Instruments, Quebec, Canada) and analyzed using WinRHIZO software (Regent Instruments). The independent experiment was repeated twice with three replicates (50 plants per replicate for seedlings and 3 plants per replicate for panicle collection).

### Immune response analysis.

The rice leaves were stained by diaminobenzidine (DAB; Biotopped) and aniline blue (Macklin), respectively, for the H_2_O_2_ accumulation and callose deposition assay. Average H_2_O_2_ and callose measurements were performed as described previously (48). Three replicates were performed for each treatment.

### RNA extraction and expression analysis.

Rice leaves (0.05 g) were collected for total RNA extraction by using a Plant Total RNA isolation kit (Sangon Biotech, China). The resulting RNA was reverse transcribed using a PrimeScript RT reagent kit with gDNA Eraser (TaKaRa, Japan). The genes expressions were analyzed by qRT-PCR, as described previously (48). The primers used for qRT-PCR are listed in Table S3. The transcript data were normalized using β-actin mRNA expression levels as the internal reference. Independent experiments were repeated twice, and all reactions were performed in triplicate.

### Protein extraction and identification.

The leaves of rice seedlings in each treatment (0.1 g) were harvested. Total protein extraction and trypsin digestion were performed according to a previous report (53). After trypsin digestion, the peptides were desalted using a Strata X C_18_ SPE column (Phenomenex) and vacuum dried. To identify endogenous rice proteins, peptides were reconstituted in 0.5 M TEAB (triethylammonium bicarbonate buffer) and processed according to the manufacturer’s protocol for the tandem mass tag kit. High-pressure liquid chromatography fractionation and liquid chromatography-tandem mass spectrometry (LC-MS/MS) analysis of the tryptic peptides were performed as described previously (54). The resulting MS/MS data were processed using the MaxQuant search engine (v.1.5.2.8). MS/MS spectra were searched against the Uniprot_OSjaponica 39947 database (63,195 sequences) concatenated with the Universal Protein Resource (UniProt) database for rice were performed as described previously (55). Independent experiments were repeated twice.

### Data analysis and bioinformatic annotation.

In the MS quantitative analysis, the intensities of multiple unique peptides of each protein were detected and are presented as the quantitative value of the corresponding proteins. The intensities of multiple peptides of each protein in the two compared samples were taken to log_2_ (so that the data conform to the normal distribution), and then we used a two-sample two-tailed *t* test method to calculates the *P* value. When the *P* value was <0.05, a change in differential expression of >1.3 is regarded as the change threshold of significant upregulation, and <1/1.3 is regarded as the change threshold of significant downregulation. The functions of the proteins were annotated based on gene ontology terms from the UniProt-GOA database. The pathway enrichment of the proteins was analyzed using the Kyoto Encyclopedia of Genes and Genomes (KEGG) database.

### Statistical analysis.

Statistical parameters are reported in the figures and figure legends. Normally, statistical analysis of the data was performed by one-way repeated-measures analysis of variance (ANOVA) using SPSS software (Chicago, IL), followed by Tukey’s *post hoc* test or Duncan’s multiple-range tests, which were used to compare the means for the multiple groups. A *P* value of <0.05 was considered statistically significant.

### Data availability.

The data that support the findings of this study are available from the corresponding author upon reasonable request.
