# Supplementary material for: Endophytic Streptomyces hygroscopicus OsiSh-2-Mediated Balancing between Growth and Disease Resistance in Host Rice
Source: mBio. 2021 Aug 10;12(4):e01566-21. doi: 10.1128/mBio.01566-21 (PMC8406269; doi:10.1128/mBio.01566-21)
Supplement: TABLE S2 [file mbio.01566-21-st002.doc]

| **TABLE S2A** The number of up-regulated DEPs in different compared groups | | | | | | |
| --- | --- | --- | --- | --- | --- | --- |
| Functional classification |  | KEGG pathway | E-M+/E-M- | E+M+/E+M- | E+M+/E-M+ | |
|  |  |  | >1.3 | >1.3 | <1/1.3 | >1.3 |
| Energy-supply pathways | Fatty acid metabolism | osa00071 Fatty acid degradation | 14 | 10 | 3 | 0 |
|  |  | osa01212 Fatty acid metabolism | 10 | 8 | 2 | 0 |
|  |  | osa00062 Fatty acid elongation | 3 | 0 | 3 | 0 |
|  |  | osa01040 Biosynthesis of unsaturated fatty acids | 6 | 5 | 1 | 0 |
|  |  | osa00072 Synthesis and degradation of ketone bodies | 3 | 1 | 2 | 0 |
|  | Glycometabolism | osa00620 Pyruvate metabolism | 18 | 11 | 3 | 0 |
|  |  | osa00010 Glycolysis / Gluconeogenesis | 27 | 8 | 12 | 0 |
|  |  | osa00040 Pentose and glucuronate interconversions | 3 | 0 | 1 | 1 |
|  | Carbon metabolism | osa01200 Carbon metabolism | 24 | 28 | 0 | 5 |
|  |  | osa00710 Carbon fixation in photosynthetic organisms | 6 | 8 | 1 | 3 |
| Energy-consuming pathways | Amino acids metabolism and protein processing | osa01230 Biosynthesis of amino acids | 35 | 25 | 5 | 0 |
|  |  | osa00380 Tryptophan metabolism | 8 | 6 | 1 | 0 |
|  |  | osa00480 Glutathione metabolism | 7 | 7 | 1 | 4 |
|  |  | osa00350 Tyrosine metabolism | 5 | 4 | 2 | 0 |
|  |  | osa00250 Alanine, aspartate and glutamate metabolism | 2 | 6 | 0 | 2 |
|  |  | osa00280 Valine, leucine and isoleucine degradation | 12 | 13 | 3 | 0 |
|  |  | osa00310 Lysine degradation | 6 | 4 | 1 | 0 |
|  |  | osa03010 Ribosome | 46 | 20 | 15 | 0 |
|  |  | osa04141 Protein processing in endoplasmic reticulum | 25 | 6 | 7 | 0 |
|  | Defense response | osa04144 Endocytosis | 19 | 2 | 7 | 0 |
|  |  | osa04146 Peroxisome | 13 | 13 | 1 | 1 |
|  |  | osa04016 MAPK signaling pathway - plant | 2 | 1 | 1 | 0 |
|  |  | osa01110 Biosynthesis of secondary metabolites | 58 | 71 | 1 | 3 |
|  |  | osa00592 alpha-Linolenic acid metabolism | 22 | 26 | 1 | 0 |
|  |  | osa00900 Terpenoid backbone biosynthesis | 5 | 3 | 3 | 1 |
|  |  | osa00940 Phenylpropanoid biosynthesis | 20 | 5 | 9 | 1 |
|  |  | osa04626 Plant-pathogen interaction | 12 | 5 | 3 | 0 |
| Total |  |  | 411 | 296 | 89 | 21 |

| **TABLE S2B** The number of down-regulated DEPs in different compared groups | | | | | | |
| --- | --- | --- | --- | --- | --- | --- |
| Functional classification | | KEGG pathway | E-M+/E-M- | E+M+/E+M- | E+M+/E-M+ | |
|  |  |  | <1/1.3 | <1/1.3 | <1/1.3 | >1.3 |
| Energy fixation and nutrient absorption | Photosynthesis | osa00195 Photosynthesis | 32 | 17 | 0 | 11 |
|  |  | osa00860 Porphyrin and chlorophyll metabolism | 7 | 0 | 0 | 0 |
|  | Carbon fixation and nitrogen metabolism | osa01200 Carbon metabolism | 34 | 3 | 0 | 5 |
|  |  | osa00710 Carbon fixation in photosynthetic organisms | 22 | 5 | 1 | 3 |
|  |  | osa00630 Glyoxylate and dicarboxylate metabolism | 20 | 5 | 5 | 3 |
|  |  | osa00030 Pentose phosphate pathway | 8 | 0 | 0 | 1 |
|  |  | osa00040 Pentose and glucuronate interconversions | 3 | 0 | 1 | 1 |
|  |  | osa00051 Fructose and mannose metabolism | 5 | 0 | 0 | 1 |
|  |  | osa00910 Nitrogen metabolism | 6 | 0 | 0 | 3 |
| Energy-consuming pathways | Oxidation-reduction process | osa00190 Oxidative phosphorylation | 3 | 7 | 1 | 2 |
|  |  | osa00591 Linoleic acid metabolism | 4 | 1 | 0 | 4 |
|  |  | osa00053 Ascorbate and aldarate metabolism | 4 | 2 | 1 | 3 |
|  |  | osa00480 Glutathione metabolism | 5 | 9 | 1 | 4 |
|  |  | osa04146 Peroxisome | 7 | 0 | 1 | 1 |
|  | Amino acids metabolism | osa00260 Glycine, serine and threonine metabolism | 8 | 0 | 0 | 0 |
|  | Biosynthesis of secondary metabolites | osa00360 Phenylalanine metabolism | 4 | 3 | 1 | 0 |
|  |  | osa00900 Terpenoid backbone biosynthesis | 5 | 2 | 3 | 1 |
| Total |  |  | 177 | 54 | 15 | 43 |
