# Supplementary material for: Endophytic Streptomyces hygroscopicus OsiSh-2-Mediated Balancing between Growth and Disease Resistance in Host Rice
Source: mBio. 2021 Aug 10;12(4):e01566-21. doi: 10.1128/mBio.01566-21 (PMC8406269; doi:10.1128/mBio.01566-21)
Supplement: TABLE S3 [file mbio.01566-21-st003.doc]

| **Marker or gene** | **Locus number or accession number** | **Forward primer (5’-3’)** | **Reverse primer (5’-3’)** |
| --- | --- | --- | --- |
| *OsRbohB* | LOC_Os09g26660 | CTGGACAGGACCAAGAGCAG | ATCTTGAACGGAGCAGCACA |
| *OsActin* | LOC_Os03g50885 | GAGTATGATGAGTCGGGTCCAG | ACACCAACAATCCCAAACAGAG |
| *OsUbq* | LOC_Os03g13170 | TTCTGGTCCTTCCACTTTCAG | ACGATTGATTTAACCAGTCCATGA |
| *MoPot2* | KY412197 | ACGACCCGTCTTTACTTATTTGG | AAGTAGCGTTGGTTTTGTTGGAT |
| *ShRpoA* | OsiSh-2GM005295 | GCAAGGGCAAGCTGGAGATG | ATGGAGTCGACCGGGATACG |
| *ADH2* | XM_015761366 | CTGGTGCTTCACGGATTATTGG | GGCTTGTCGTAATCTTTTGGGTT |
| *PGAM-i* | XM_015784345 | CGTGTTCGTGTCCCAAAGATCC | TCCAGAGCGGTTTCCATTCC |
| *KSL11* | LC030235 | GCAGGTGGTGTGAGGAATAAC | GCCGTGTCGTATGAAGATGG |
| *Rps4-30* | NC_001320 | CTATTGCTTCATCCGACCCG | GAGACCGACCCACTTCCTATCC |
| *RPS17* | XM_015759294 | CGGGGCATCTCGCTGAAG | TACGGCGGGCGGGAGTAGGT |
| *CNX* | XM_015779952 | TCTGATCCGATTGCCGCTAT | GCCTTCTCCTTCTCCTTCTCTA |
| *FDH* | XM_015776894 | TCACCATCTCCGTCTACATCAT | GGCTATCCTCGTCGAACTTG |
| *PBZ1* | LOC_Os12g36880 | GTGGGAAGCACATACAAGACC | AGGGTGAGCGACGAGGTAG |
| *LAP1* | XM_015763516 | AGGCTTACACTTGCTGATGCTT | GCGACTTCCTTGTCCAGTTCAT |
| *RABA2a* | XM_015783679 | CGTGAGCAAGCCCACAACC | CCTGCCCCGCCTTCATCT |
| *AGD9* | XM_015774937 | TCGGCGTCCACATCACCTT | TGCCACCATCTGTCCACCC |
| *DRP2a* | XM_015771175 | GTGCTGCCGACGGGTGAGA | GCCGACACCTGCTGCGACT |
| *atpE* | NC_001320 | GGATGTAAAGCCAAGCA | TCTCAGGTCCTAAATAAACG |
| *LFNR2* | XM_015769712 | GGCTGCGGAGGTGACTACT | ATGACGCCGATGGACTGC |
| *FDX3* | XM_015774279 | CCGAGACCGCTGGAGTA | CGGACTTAGGGTAGGAGACA |
| *GLN2* | XM_015779836 | TGGACCTAGTGTCGGTATTG | CTCTTGGTGCTGTAGTTTGTG |
| *CAs* | XM_015761752 | AACTCATCGTGGTGATTGGC | CGCATTGGTCATCGAAAGG |
| *ALDOA* | AC128643 | CATCCTCCTCAAGCCAAGCAT | GCCCACCCGACAAGAACAT |

**TABLE S3** RT-qPCR primers used in this study
